# Compressive Strength of Conventional Glass Ionomer Cement Modified with TiO_2_ Nano-Powder and Marine-Derived HAp Micro-Powder

**DOI:** 10.3390/ma14174964

**Published:** 2021-08-31

**Authors:** Ana Ivanišević, Valentina Brzović Rajić, Ana Pilipović, Matej Par, Hrvoje Ivanković, Anja Baraba

**Affiliations:** 1 School of Dental Medicine, University of Zagreb, Gundulićeva 5, 10000 Zagreb, Croatia; aivanisevic@sfzg.hr (A.I.); mpar@sfzg.hr (M.P.); baraba@sfzg.hr (A.B.); 2 Faculty of Mechanical Engineering and Naval Architecture, University of Zagreb, Lučićeva 5, 10000 Zagreb, Croatia; ana.pilipovic@fsb.hr; 3 Faculty of Chemical Engineering and Technology, University of Zagreb, Marulićev trg 19, 10000 Zagreb, Croatia; hivan@fkit.hr

**Keywords:** glass ionomer cement, compressive strength, titanium dioxide, hydroxyapatite

## Abstract

The aim of this research was to investigate the compressive strength (CS), breaking strength (BS), and compressive modulus (CM) of conventional glass ionomer cement (GIC) modified with TiO_2_ nano particles, marine-derived hydroxyapatite (md-HAp) microparticles (<45 µm), and a combination of TiO_2_ NP and md-HAp particles. The materials used in the study were conventional GIC Fuji IX GP Extra (GC Corporation, Tokyo, Japan), TiO_2_ powder P25 (Degussa, Essen, Germany), and HAp synthesized from cuttlefish bone and ground in a mortar to obtain md-HAp powder. md-HAp was characterized using FTIR and SEM analysis. There were four groups of GIC samples: (i) Fuji IX control group, (ii) powder modified with 3 wt% TiO2, (iii) powder modified with 3 wt% HAp, and (iv) powder modified with 1.5 wt% TiO2 + 1.5 wt% HAp. Measurements were performed in a universal testing machine, and CS, BS, and CM were calculated. Statistical analysis was performed using ANOVA and Tukey’s tests. CS, BS, and CM differed significantly between the Fuji IX control group and all experimental groups while differences between the experimental groups were not statistically significant. The addition of TiO_2_ NP, md-HAp micro-sized particles, and a combination of TiO_2_ and md-HAp reduced the CS, BS, and CM of conventional GICs when mixed at the powder/liquid (p/l) ratio recommended by the manufacturer.

## 1. Introduction

Glass ionomer cements (GICs) were invented by Wilson and Kent more than 50 years ago and introduced into dentistry as translucent cements [1]. GICs are two-component materials containing a fluoro-aluminosilicate powder and an aqueous solution of organic polyalkenoic acids, predominantly polyacrylic acid. After mixing the two components, GICs are set by the neutralization reaction between the polyalkenoic acids and metal oxides from fluoro-aluminosilicate glass particles to form insoluble polysalts and water [2]. The cross-linking of the polyacrylate chains with metal ions (calcium and aluminum) forms a matrix in which glass particles are enclosed [2]. The cross-linking ensures the strength, stiffness, and insolubility of the cements, and is influenced by the characteristics of the GIC material’s components (polyacid and powder composition), powder-to-liquid ratio, and the environmental conditions in which the setting occurs [3,4]. The favorable properties of GIC materials are their ability to chemically bind to enamel and dentin, anticariogenic activity through the release of fluoride ions, and biocompatibility. These properties have made them widely accepted for use in clinical dentistry as luting materials, cavity liners, and restorative materials [2,3]. Besides in dentistry, GICs have been found useful even in neuro-otological surgeries and reconstruction surgeries of the maxillofacial region due to their biocompatibility and adhesion to bone structure [5,6,7].

Despite these favorable characteristics, GICs do have certain disadvantages, including inferior physical properties—relatively low compressive strength, brittleness, and poor wear characteristics [8]. To improve their inferior physical properties, GICs have undergone various modifications, including increased powder/liquid (p/l) ratio, different particle sizes, and modification with resin [9,10]. Moreover, several materials have been proposed as GIC additives to enhance physical properties, including titanium dioxide (TiO_2_), hydroxyapatite (HAp), and others [11,12,13]. HAp is a major calcified component of bone and hard dental tissues. When added to the GIC powder, HAp was reported to interact with carboxylate groups of polyacids, thus improving cross-linking and hardness of the cement [14,15]. Marine-derived HAp was shown to be biocompatible, readily available, and low cost [16]. Furthermore, titanium dioxide (TiO_2_) is a chemically stable, biocompatible inorganic additive exhibiting antibacterial properties. TiO_2_ nano particles (NP) were shown to have antibacterial effects and to potentially reinforce GICs [11,17]. The microhardness, flexural and compressive strength, and antibacterial activity of TiO_2_ NP-modified GICs were found to be improved without interfering with adhesion to mineralized dental tissues and with fluoride release. The improvement was more pronounced at 3% (*w*/*w*) than at 5% and 7% (*w*/*w*) [11]. Although there are some concerns about the potentially cytotoxic effects of TiO_2_ NP, its toxicity is generally considered to be low [18,19]. The combination of HAp and TiO_2_ has been used for coating titanium implants with improved osteogenic activity around the implants [20]. According to the available literature, there have been no studies dealing with the mechanical properties of GICs after simultaneous addition of HAp and TiO_2_ NP.

Based on previous reports, we expected that supplementing TiO_2_ NPs and HAp micro-sized particles into GIC would enhance the mechanical properties. The objective of this research was to investigate the compressive strength (CS), breaking strength (BS), and compressive modulus (CM) of conventional GIC (Fuji IX) modified with 3% (*w*/*w*) TiO_2_ NP, 3% (*w*/*w*) md-HAp, and combined 1.5% (*w*/*w*) TiO_2_ + 1.5% (*w*/*w*) md-HAp.

## 2. Materials and Methods

**Figure 1:**
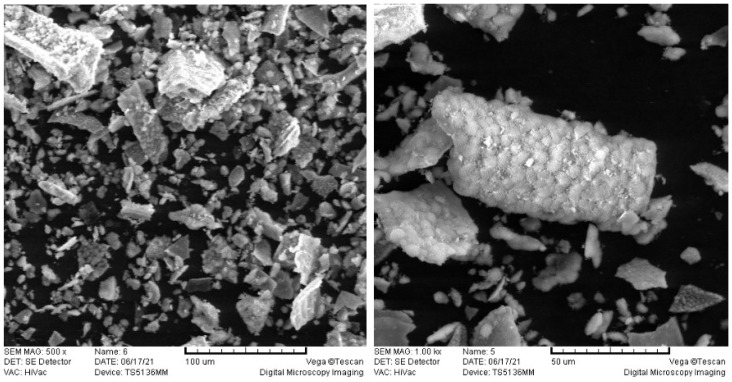
SEM images of md-HAp powder at magnifications of 500× and 1000×. md-HAp was ground in a mortar and sifted through a 45 μm sieve resulting in the powder composed of angular particles <45 µm.

A commercially available conventional GIC was used in this study: Fuji IX GP Extra (GC Corporation, Tokyo, Japan). Commercial TiO_2_ powder P25 was also used (Degussa, Essen, Germany). The specifications of the TiO_2_ were: mineral composition 85% rutile and 15% anatase, purity 99.9%, average size of primary particles 20 nm, molar mass 79.87 g/mol, density 4.26 g/cm^3^. Hydroxyapatite powder was synthesized from cuttlefish bone [21], ground in a mortar and sifted through a 45 μm sieve, resulting in the HAp powder with particles <45 µm (Figure 1).

### 2.1. Characterization of md-HAp Using Fourier Transform Infrared (FTIR) Spectroscopy

Fourier-transform infrared (FTIR) spectroscopy was performed on the marine-derived HAp powder and an analytical grade HAp powder (Merck, Darmstadt, Germany). The diamond attenuated total reflectance (ATR) accessory of Nicolet iS50 FTIR spectrometer (Thermo Fisher Scientific, Waltham, MA, USA) was covered with 0.05 g of the powder, which was gently distributed and pressed on the diamond ATR crystal using a modified accessory press. FTIR spectra were collected in absorbance mode using a mercury-homogenous cadmium-telluride detector (spectral range: 3500–400 cm^−1^, resolution: 4 cm^−1^, number of scans per spectrum: 20). The assignments of FTIR spectral bands were performed according to Figueiredo et al. [22].

### 2.2. Preparation of Samples and Determining Mechanical Properties

The fluoro-aluminosilicate glass powder of commercial GIC, HAp powder, and TiO_2_ powder were manually mixed with a mortar and pestle for 10 min to obtain as homogenous a distribution as possible of HAp and/or TiO_2_ in the Fuji IX fluoro-aluminosilicate glass powder. The prepared powders were then mixed with the liquid component by plastic spatula, according to the manufacturer’s instructions, at a recommended powder/liquid ratio of 3:6. Four groups were prepared—a control group without any particles added and three experimental groups in which the powder was modified with 3 wt% TiO_2_, 3 wt% HAp, and 1.5 wt% TiO_2_ + 1.5 wt% HAp.

For CS, BS, and CM testing, cylinder-shaped samples were made for each group (*N* = 4). After mixing, the material was instilled into silicone moulds (4 mm diameter × 8 mm height) using a syringe (Centrix, Shelton, CT, USA). Polyester strips were placed on both sides of the mould, and the material was gently compressed. The samples were left for 1 h to harden. The samples were then removed from the silicone mould and kept in deionized water for a week. After complete setting, the samples were polished in steel moulds on a grinder-polisher (Buehler, IL, USA) using 500-grit carbide paper under continuous water rinsing. Final dimensions of the samples for compressive strength testing were 4 mm diameter and 6 mm height.

**Figure 2:**
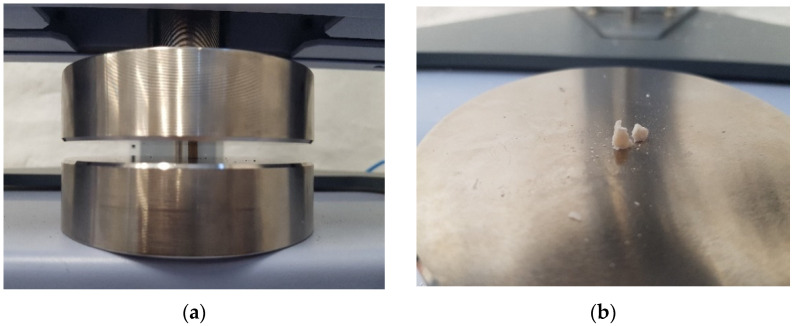
The testing procedure (**a**) and the specimen after testing (**b**).

The measurements were performed according to ISO specification 7489:1986 [23] at a speed of 0.75 mm/min, room temperature of 22 °C, and relative humidity of 45% (Figure 2). The CS of each specimen, expressed in N/mm^2^, was calculated using the equation:

(1) $$CS=\frac{4\cdot F}{\pi \cdot d^{2}}$$

where *F* (N) is the max. force and *d* (mm) is the diameter of the specimen.

Compression breaking strength was calculated by the same equation; only the value of the force at the moment of breaking of the test specimen was used in the calculation.

Compression modulus *E*_c_ is calculated according to the equation:

(2) $$E_{C}=\frac{\sigma }{\varepsilon }=\frac{\sigma_{2}-\sigma_{1}}{\varepsilon_{2}-\varepsilon_{1}}$$

where *E*_c_ (N/mm^2^) is compression modulus, *σ* (N/mm^2^) is stress, and *ε* (%) is strain.

In this testing, stress values were taken from *σ*_2_ = 40 N/mm^2^ and *σ*_1_ = 20 N/mm^2^, and for these values, their deformations were read.

The data were statistically analysed using descriptive analysis, a one-way ANOVA, and a post-hoc Tukey’s test at a level of significance *p* = 0.05. Normality of distribution was tested using the Shapiro-Wilk test. Levene’s test was used for equality of variances testing, and in the case of non-homogeneous variances, the Welch’s variant of ANOVA test was used.

## 3. Results

### 3.1. FTIR Analysis of md-HAp

**Figure 3:**
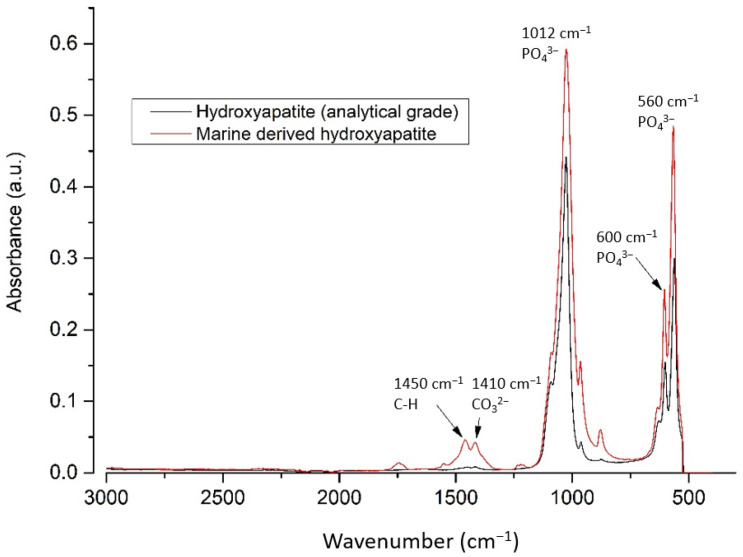
Spectra of analytical grade HAp and md-HAp showed a high degree of concordance.

Spectra of md-HAp powder and pure hydroxyapatite were recorded and compared. The bands at 560, 600, and 1012 cm^−1^ correspond to the vibrations of the PO_4_ group and are characteristic of hydroxyapatite. Additionally, the bands at 1450 and 1410 cm^−1^ in md-HAp correspond to the organic part (CH_2_) and the carbonate group (Figure 3). FTIR spectra thus showed that the md-HAp powder consisted of carbonated hydroxyapatite with some organic (protein) content [22].

### 3.2. Mechanical Properties of the GIC Samples

The dimensions of the samples (4 mm × 6 mm) between the four experimental groups did not significantly differ (ANOVA, *p* > 0.05 for diameter and height).

**Figure 4:**
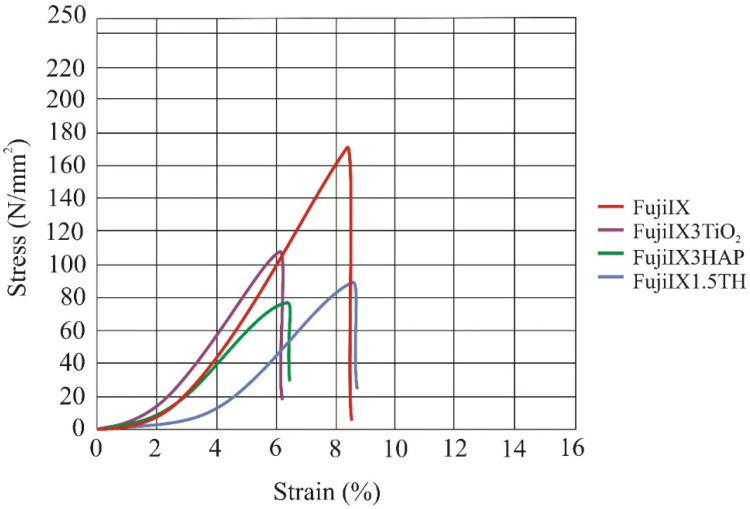
Compression stress–strain diagram. The mean values for the four groups of materials (*N* = 6) are shown in the diagram. The control group in which Fuji IX was not modified exhibited the highest compressive stress and compressive strength. Legend: FujiIX is a control group, FujiIX3TiO_2_ is material with 3% of TiO_2_, FujiIX3HAP is material with 3% HAp and FujiIX1.5TH material with 1.5% of TiO_2_ and 1.5% of HAp.

The compression stress-strain diagram for the four groups of samples is shown in Figure 4. The diagram shows mean curves (mean values are shown in Table 1) per batch. The force and displacement were recorded every 0.1 s until the test specimen broke. After that, the strength and strain values were calculated from these data according to Equations (1) and (2), and the mean values were calculated. At smaller load, the stress-strain curve is flatter and has higher displacement (initial loading) until the surface of the sample gets aligned to the surface of the testing machine’s jaw.

The results for compressive strength, breaking strength, and compressive modulus for the Fuji IX control and the three experimental groups are given in Table 1.

Statistical analysis showed that the differences between the four groups were statistically significant for all mechanical properties measured (ANOVA test). Individual comparisons between the groups using Tukey’s test showed that the compressive strength, breaking strength, and compressive modulus differed significantly between Fuji IX control group and all experimental groups (modified with 3 wt% HAp, 3 wt% TiO_2,_ and 1.5 wt% HAp + 1.5 wt% TiO_2_), while the differences between the experimental groups were not statistically significant. The mean values of deformation at break differed between the groups (*p* = 0.03),

## 4. Discussion

The results of the present study revealed that the modification of a conventional GIC powder with nano particles of TiO_2_ and micro particles of md-HAp (<45 µm) did not improve compressive strength, compressive modulus, and breaking strength. On the contrary, the tested mechanical properties deteriorated, and in some cases, significantly. The hypotheses assumed enhanced compressive strength, compressive modulus, deformation break, and breaking strength among the modified materials, and were thus rejected.

Compressive and flexural tests are used to simulate the stress applied to materials used in clinical dentistry. Most mastication forces are compressive in nature, and compressive tests reveal the critical value at which the material breaks/fails during mastication. The minimum value necessary to resist the masticatory forces in the posterior teeth would be 125 MPa, while it would be 100 MPa for primary dentition [24,25]. This implies that the samples from the experimental groups with modified FAS powders do not have sufficient compressive strength to resist forces in the posterior region.

The addition of 3% (*w*/*w*) TiO_2_ significantly reduced the compressive strength of Fuji IX as well as the breaking strength and compressive modulus. This result is not in accordance with the results of previous studies [11,18]. The reason for this was perhaps because the p/l ratio was unchanged in the experimental powder modified with nanoparticles of TiO_2_. In a previous study, the original p/l ratio was reduced when the original FAS powder was replaced with nano FAS powder because of the low bulk density of the nano-sized powder [26]. Although in our study the original GIC powder was modified with only 3 wt% TiO_2_ NP, the reduction of mechanical properties could be explained by the overall lower bulk density of the modified powder. Moreover, since p/l ratio was shown to influence mechanical properties [3], the intention was to have the p/l ratio the same in all groups so that the influence of the powders’ composition on mechanical properties would be the sole variable. TiO_2_ NP between the larger FAS glass particles can also interact with polyacrylic acid [27], but it might be that the carboxylic groups were insufficient to react with the high surface to volume ratio TiO_2_ particles, so the interfacial bonding was weaker between the particles and the cement matrix. The unfavourable results after mixing TiO_2_ NP might be different if nanotubes were used, similar to the study by Cibim et al. [28].

Modification with 3% (*w*/*w*) md-HAp powder also reduced mechanical properties. CS was even lower than with modification with 3% (*w*/*w*) TiO_2_ NP. It was previously suggested that the HAp particles dispersed in FAS react with polyalkenoic acid similarly to HAp from dentin and enamel: the ionic attraction between carboxyl groups from GIC and the calcium ions in the HAp of enamel and dentine results in the displacement of calcium and phosphate ions from the HAp and the formation of an ion-exchange layer of calcium and aluminium phosphates and polyacrylates at the interface [2,29,30,31]. These additional matrix layers should improve mechanical properties. Since this was not the case in the present study, apparently these additional intermediate strengthening ionic attractions did not occur. Perhaps the HAp micro-sized particles were too big (<45 µm). Indeed, larger particles have a relatively smaller specific surface area, and there are fewer metal ions from the modified powder leaching into the ion exchange layer to form bonds. Furthermore, the wide distribution of particle sizes in GIC powder ensures a high packing density, and for optimal strength, the particles should be neither too fine nor too large [32,33]. The original Fuji IX powder contains angular particles whose size ranges from 0.3 to 200 μm, 36.91% are <5.0 µm and 51.72% are of sizes from 5.0 to 50 µm [26,33]. The addition of HAp particles <45 µm changed the ratio between the larger and fine particles, lowering packing density and negatively affecting mechanical properties. Furthermore, besides the size and distribution of the particles, the p/l ratio influences the strength. Experimental cements modified with HAp micro-sized particles had high bulk density and were oversaturated at 3:6 p/l ratio, compromising ionic interaction between HAp and carboxylic groups and cross-linking. In short, fewer ions and bonds and a higher number of unreacted particles could explain the lower strength of the experimental cements in the present study.

Moreover, the tested mechanical properties might have been reduced due to the protein content in md-HAp and carbonated HAp. This might imply that the synthetic HAp of high purity should be preferred as an additive to FAS powder, rather than the HAp derived from fish waste, although HAp and collagen derived from fish waste have shown promising results in bone tissue engineering [16].

Since GICs are also used in reconstruction surgeries, along with improved mechanical properties, the addition of components favouring osteointegration would be desirable. TiO_2_ and HAp coatings improved osteointegration of titanium implants [20]. However, the present study suggests that the combination of 1.5% (*w*/*w*) TiO_2_ NP and 1.5% (*w*/*w*) HAp did not improve compressive strength, breaking, and compressive modulus when added to Fuji IX powder, most likely due to the same reason pointed out earlier—that the p/l ratio of 3:6 was not optimal for the cement with the powder modified with the combination of the two particles.

Manual spatulation of the modified powders was harder than in the case of control samples. The recommended p/l ratio was harder to achieve, especially in the case of modification with TiO_2_ NP, due to the lower bulk density of the powder. It was harder to manipulate with the mixed cement, leading to more air inclusions, which also reduced mechanical properties. Hand mixing could be taken as a limitation of the present study. However, compressive strength values of control samples are in the range of the reported values for Fuji IX [34], so we cannot attribute the lower results in the experimental groups solely to the imperfection of manual mixing since all the samples were mixed in the same manner.

## 5. Conclusions

Combined and individual incorporation of TiO_2_ NP and md-HAp micro-sized particles into the powder of conventional GIC Fuji IX did not result in improved compressive strength, breaking strength, and compressive modulus. This could be attributed to inadequate interaction between the added particles and the GIC matrix, most likely due to an inadequate p/l ratio that left many particles unreacted.

## Figures and Tables

**Table 1:** Mean values and standard deviations (sd.) of compressive strength, breaking strength, deformation at break, and compressive modulus for the four Fuji IX groups.

Mechanical Property	0% TiO_2_ 0% HAp		3% TiO_2_		3% HAp		1.5% TiO_2_ 1.5% HAp		ANOVA
	Mean	sd.	Mean	sd.	Mean	sd.	Mean	sd.	*p*
Compressive strength (N/mm^2^)	172.71	(17.15)	109.23	(14.72)	78.52	(15.49)	91.01	(10.85)	<0.0001 ^a^
Breaking strength (N/mm^2^)	170.25	(18.45)	108.26	(14.57)	75.70	(16.02)	86.59	(12.76)	<0.0001 ^a^
Deformation at break (%)	8.50	(1.49)	6.17	(0.61)	6.41	(0.51)	8.64	(1.95)	0.03
Compressive modulus (N/mm^2^)	2955.91	(98.30)	2343.39	(195.60)	1747.29	(268.45)	1886.06	(267.68)	<0.0001 ^a^

^a^ Difference between control group and experimental groups with modified powder was statistically significant.

## Data Availability

The data that support the findings of this study are available from the corresponding author upon request.
